# Hhatl Reduces Apoptosis of Müller Cells in Diabetic Retinopathy by Relieving Endoplasmic Reticulum Stress

**DOI:** 10.1155/ije/6615457

**Published:** 2025-10-02

**Authors:** Jie Qin, Yaling Wang, Hui Cao, Bing Liu, Xiangwen Kan, Lei Wu

**Affiliations:** ^1^Department of Ophthalmology, Rizhao Central Hospital, Rizhao 276800, Shandong, China; ^2^Chronic and Special Critical Diseases Clinic, Qingdao Municipal Hospital, Qingdao 266000, Shandong, China; ^3^Department of Otorhinolaryngology, Rizhao Central Hospital, Rizhao 276800, Shandong, China; ^4^Department of Stomatology, Rizhao Central Hospital, Rizhao 276800, Shandong, China

**Keywords:** ATF6, diabetic retinopathy, endoplasmic reticulum stress, Hhatl, Müller cells

## Abstract

**Background:**

Diabetic retinopathy (DR), a microvascular disease, also involves retinal neurodegeneration. Müller cells exert an important role in the retina, and their destabilization and reduction affect the physiological function of the retina. To investigate the effect and mechanism of hedgehog acyltransferase-like (Hhatl) on Müller cells in DR.

**Methods:**

The differentially expressed genes (DEGs) in Müller cells of DR rats were first analyzed by single-cell transcriptomics techniques (scRNA-seq). Regulating Hhatl expression, cell viability was detected using cell counting kit-8 (CCK-8) assay; apoptosis was detected by terminal deoxynucleotidyl transferase nick-end labeling (TUNEL); the expression of B-cell lymphoma 2 (Bcl2), Bcl2-associated X protein (Bax), activating transcription factor 6 (ATF6), C/EBP homologous protein (CHOP), and glucose-regulated protein 78 (GRP78) was assessed by immunofluorescence; and Ca^2+^ concentration was determined by fluorescence quantification to observe the effect and mechanism of Hhatl on Müller cells of the high glucose (HG)-treated rats. Finally, the results of the cell assays were verified in male 6-week-old Zucker (fa/fa) diabetic fatty (ZDF) rats. Viral vectors expressing Hhatl were injected into the vitreous of ZDF rats, and apoptosis and endoplasmic reticulum stress (ERS)-related indices in rat retinal cells were detected using immunofluorescence.

**Results:**

scRNA-seq analysis revealed that Hhatl was low-expressed in Müller cells of DR rats. In vitro assays confirmed that upregulation of Hhatl could increase rMC-1 Bcl2 expression, decrease Bax expression, and reduce apoptosis in HG environments. In addition, Hhatl did downregulate ATF6 expression, decrease CHOP and GRP78 levels, and reduce Ca^2+^ concentration. Animal assays showed that Hhatl overexpression in the vitreous of ZDF rats did elevate Bcl2 level, decrease Bax expression, and reduce ATF6, CHOP, and GRP78 levels, which alleviated ERS in the retina of ZDF rats.

**Conclusion:**

Hhatl reduces apoptosis of Müller cells in DR by alleviating ATF6-related ERS signaling.

## 1. Introduction

With the development of the economy and the improvement of people's living standards, high-sugar and high-fat diets appear more and more frequently in people's lives, and diabetes is increasingly becoming an important disease that threatens human health. Diabetes is a chronic metabolic disease characterized by hyperglycemia. Prolonged hyperglycemia can lead to various complications that seriously affect patients' quality of life and lifespan [1]. Diabetic retinopathy (DR) is one of the major complications of diabetes, and patients with DR may become blind due to diabetic macular edema (DME) and/or proliferative DR (PDR), causing much inconvenience in daily life [2]. Numerous studies have demonstrated that inflammation, oxidative stress, and pyroptosis are associated with DR progression [3–5]. So far, there are no methods that can cure DR. Therefore, it is crucial for therapeutic approaches and drug development to search for new target molecules and potential mechanisms of DR.

Endoplasmic reticulum (ER) is one of the most important intracellular organelles with a large number of biological functions, including protein synthesis, folding and transport, lipid synthesis, and calcium (Ca^2+^) storage and gated release. These processes are regulated by the unfolded protein response (UPR), whose prolonged activation or dysregulation causes ER stress (ERS) and ultimately cell death. In addition, these processes are implicated in the pathogenesis of many human diseases, including DR [6]. Müller cells are the primary glial cells spanning the entire retina and exert an irreplaceable role in the retina. For instance, Müller cells can control retinal metabolism and nutrient supply, regulate blood flow, and maintain the blood-retinal barrier (BRB) [7]. Hyperglycemia can induce ERS in Müller cells through multiple pathways. Excessive glucose metabolism triggers hyperactivity of the polyol pathway, abnormal activation of the protein kinase C (PKC) pathway, and accumulation of advanced glycation end products (AGEs). All of these will disrupt the protein folding environment in the ER [8]. High glucose (HG) can cause excessive reactive oxygen species (ROS) production in intracellular mitochondria and exacerbate ER oxidative damage through pathways such as NADPH oxidase, ultimately leading to the accumulation of unfolded proteins [9]. In diabetes, oxidized low-density lipoprotein (ox-LDL) and ox-LDL immune complexes (ox-LDL-ICs) can mediate stress responses in Müller cells through the cluster of differentiation 36 (CD36) receptor [10]. In the diabetic state, dysfunction of Müller cells can lead to the disruption of the BRB. Under stress conditions, Müller cells will over-secrete vascular endothelial growth factor (VEGF), which promotes the proliferation of retinal vascular endothelial cells (RVECs) and the formation of abnormal neovascularization, thereby exacerbating vascular leakage [11, 12]. Meanwhile, HG can induce reactive gliosis in Müller cells [13]. Activated Müller cells also release chemokines such as interleukin-1β (IL-1β) and interleukin-8 (IL-8) to recruit leukocytes and macrophages, thereby forming an “inflammation-injury” cycle and further exacerbating vascular and neural damage [2]. In DR, increased nuclear translocation of glyceraldehyde 3-phosphate dehydrogenase (GAPDH) is involved in ERS-induced apoptosis of Müller cells [14]. The reduction in Müller cell numbers disrupts the retinal microenvironmental homeostasis, accelerating the progression of DR [15]. Currently, researchers have been committed to developing therapeutic methods to alleviate ERS in Müller cells. Li et al. have found that Semaphorin 3A could alleviate ERS in primary mouse Müller cells under HG environments [16]. Ren et al. have used liraglutide to activate the p-Erk pathway and attenuate oxidative stress and ERS, thereby reducing HG-induced apoptosis in Müller cells [17]. These studies provide new ideas for the treatment of DR.

Hedgehog acyltransferase-like (Hhatl) was originally reported as a cardiac-specific gene highly expressed in heart and skeletal muscle [18]. In zebrafish, deletion of hhatla (a homologue of Hhatl) results in cardiac defects, and suppression of hhatla promotes the expression of cardiac hypertrophy markers, leading to ventricular hypertrophy [19]. A recent study has shown that downregulation of Hhatl in rat cardiomyocytes did elevate the expression of hypertrophic markers and ROS, ultimately causing cardiac hypertrophy and mitochondrial dysfunction [20]. Shi et al. have found that Hhatl promotes autophagy by associating with microtubule-associated protein 1 light chain 3 (LC3) through the LC3-interacting region (LIR) and ameliorates ERS through autophagy activation to relieve cellular stress [21]. In this study, Hhatl was found to be low-expressed in DR rat Müller cells by single-cell transcriptomics (scRNA-seq) and differential gene expression analysis, but its effects in DR remain unknown. Therefore, the present study hypothesizes that overexpression of Hhatl can alleviate ERS in Müller cells and reduce the apoptosis rate via activating transcription factor 6 (ATF6) signaling pathway inhibition, thereby mitigating the progression of DR. To verify this hypothesis, the present study will simulate the natural course of human DR using Zucker (fa/fa) diabetic fatty (ZDF) rats (a spontaneous type 2 diabetes model). Additionally, primary rat Müller cells will be cultured under HG conditions. Both in vivo and in vitro approaches will be employed to investigate the regulatory role of Hhatl in DR, aiming to clarify its potential as a DR intervention target and associated molecular pathways.

## 2. Materials and Methods

### 2.1. Data Analysis

The GSE209872 dataset from the Gene Expression Omnibus (GEO) (https://www.ncbi.nlm.nih.gov/geo/) was subjected to single-cell transcriptomics (scRNA-seq) using the R package Seurat (v4.3.1). The dataset included a total of 1 nondiabetic control rat retina sample and 3 rat DR samples (one control normal sample in the GSE209872 dataset exhibited abnormal data integrity and was therefore excluded). In the data preprocessing stage, to exclude some low-quality cells as well as low-expressed genes, the following thresholds were set: (1) the number of genes per cell ranged from 200 to 6000; (2) the expression rate of mitochondrial genes was less than 25% in each cell. The batch effect of the two samples was successfully removed via the Harmony algorithm, obtaining a consistent dataset containing 43,527 cells and 16,330 genes. Subsequently, the scRNA-seq dataset was standardized using the NormalizeData function, and 2000 highly variable genes (HVGs) were identified using the vst method in the FindVariableFeatures function.

The data after normalization were subjected to principal component analysis (PCA), and the first 30 PCs were selected for subsequent in-depth analysis. Based on these 30 PCs, cells were clustered with the FindClusters function, obtaining 26 cell populations. The original dataset text-related cell marker genes and common cell markers were extracted, and 12 cell populations were annotated by the expression of the cell marker genes in different cell populations. The top 20 significantly differentially expressed genes (DEGs) were screened by carefully selecting the fold change (FC) of Müller cell genes. After obtaining the data of Müller cells, an in-depth dimensionality reduction clustering analysis was performed to re-annotate Müller cell subsets.

### 2.2. Animals and Assay Design

Specific pathogen-free (SPF) grade male 6-week-old ZDF rats (*n* = 36, body weight 200 ± 20 g) were purchased from Charles River Laboratories (Waltham, MA, USA). The rats were fed with the Purina 5008 diet and had free access to food and water. Due to a homozygous mutation of the fa gene (chromosome 5q31), ZDF rats have a deficiency in leptin receptor function, manifesting as congenital obesity and insulin resistance. The animals were housed in cages, with 3 rats per cage, and all were housed in an environment with a temperature of 22 ± 2°C, humidity of 50 ± 10%, and a 12 h light/dark cycle. The bedding was sterilized corn cobs, and cage positions were randomly rotated weekly according to a random number table.

Viral vector adeno-associated virus serotype 2 (AAV2) and viral vector expressing Hhatl (AAV2-Hhatl) were purchased from Obio Biotechnology (Shanghai, China). ZDF rats with fasting blood glucose levels > 16.7 mmol/L were grouped using a simple randomization method. Random numbers (1–36) corresponding to the number of rats were generated in Excel, sorted by the size of the random numbers, and divided into three groups of 12 rats each (*n* = 12). Group 1 (numbers 1–12) was the control group, group 2 (numbers 13–24) was the AAV2 group (AAV2), and group 3 (numbers 25–36) was the AAV2-Hhatl group. Intravitreal injections were performed with reference to previously published literature [22]. Viral vectors were slowly injected into the vitreous of ZDF rats using a microsyringe at weeks 18 and 22. Prior to vitreous injection, the rats were anesthetized by intraperitoneal injection of 2% sodium pentobarbital, and a mixture of 1% tropicamide and 1% phenylephrine hydrochloride was added intraocularly for pupil dilation. If the rats show postoperative agitation, butorphanol (0.1 mg/kg) will be injected subcutaneously for analgesia. The control group was intravitreally injected with 4 μL of phosphate-buffered saline (PBS). The AAV2 and AAV2-Hhatl groups were intravitreally injected with 4 μL of AAV2 or AAV2-Hhatl (1 × 10^13^ viral genomes [vg]/mL), respectively. The eyeballs were removed after CO_2_ over-inhalation was administered to the assay rats at week 24 and rapidly frozen at −80°C. All experiments involving animals were approved by the Animals Ethics Committee of OBiO Technology (Shanghai) Corp., Ltd. (Ethics approval number: IACAC-143).

### 2.3. Cell Culture and HG Treatment

The rat retinal Müller cell line (rMC-1) cells, purchased from Kerafast Inc. (Boston, MA, USA), were grown in Dulbecco's Modified Eagle's Medium (DMEM) containing 10% Fetal Bovine Serum (FBS) (Sigma, St. Louis, Missouri, USA) at 37°C and 5% CO_2_.

To obtain glucose incubation concentrations and durations consistent with subsequent assays, rMC-1 cells were incubated in DMEM containing different concentrations of glucose (5.5, 10, 20, 30, and 40 mM) for different durations (24, 48, and 72 h), with the 5.5 mM glucose group being the normal control group (NG). Cell viability was determined by the cell counting kit-8 (CCK-8) assay.

### 2.4. Immunofluorescence

Rat retinal tissues were fixed with 4% paraformaldehyde (PFA) on ice for 2 h and stepwise dehydrated in sucrose solution (2 h in 10%, 2 h in 20%, and overnight in 30%) at 4°C. After embedding with Sakura OTC compound (Tissue Tek, Torrance, CA, USA), the tissues were sectioned vertically with a cryostat (CM1950, Leica, Wetzlar, Germany). Three groups (CONTROL, AAV2, and AAV2-Hhatl) were set up for comparison in the experiment. rMC-1 cells were fixed in 4% formaldehyde for 1 h and divided into four groups for comparison: NG, HG, HG + overexpression negative control (OE-NC), and HG + OE-Hhatl. Then, retinal sections and rMC-1 cells were soaked in Tris-buffered saline containing 0.1% Triton X-100 (P0096, Beyotime, Shanghai, China) for 10 min at room temperature and closed with 5% bovine serum albumin (BSA) solution for 1 h. Next, the samples were separately incubated with anti-Hhatl (BW11052, KA&M BIO, Shanghai, China), anti-ATF6 (PA5-85935, Invitrogen, Carlsbad, California, USA), anti-Bcl2 (PA5-27094, Invitrogen, Carlsbad, California, USA), anti-Bax (SAB4502546, Sigma, St. Louis, Missouri, USA), anti-CHOP (SAB5700602, Sigma, St. Louis, Missouri, USA), and anti-GRP78 (SAB4501452, Sigma, St. Louis, Missouri, USA) overnight at 4°C and then incubated with Goat anti-Rabbit IgG (H + L) Secondary Antibody Alexa Fluor 594 (A-11037, Invitrogen, Carlsbad, California, USA) for 1 h at room temperature. Subsequently, the DAPI working solution (#C1005, Beyotime, Shanghai, China) was added dropwise for nuclear staining. All images were taken under the same settings.

### 2.5. Cell Transfection

Rat Hhatl cDNA was cloned into pcDNA3.4 to construct an overexpression vector (OE-Hhatl). Cells were inoculated onto new culture dishes with a medium free of serum and antibiotics, and plasmids were transfected into cells using Lipofectamine 3000 Transfection Reagent (L3000075, Invitrogen, Carlsbad, California, USA). Subsequent assays were performed 48 h after transfection.

### 2.6. Reverse Transcription-Quantitative Polymerase Chain Reaction (RT-qPCR)

Total RNA was extracted from rMC-1 cells and transcribed into cDNA using the SuperScript III Reverse Transcriptase Kit (18080093, Thermo Fisher Scientific, Waltham, MA, USA) according to the manufacturer's protocols. Hhatl levels were assessed in the NG, HG, HG + OE-NC, and HG + OE-Hhatl groups using qPCR SYBR Green Master Mix (Q711-02, Vazyme Biotech, Nanjing, China). Hhatl (NCBI RefSeq NM_001106868.1) forward primer: 5′-GTCCTTAACTGCTTCGGCCT-3′, reverse primer: 5′-CAAACAAGACCGCCAGTGTG-3′. β-actin (NCBI RefSeq NM_031144.3) was used as an internal reference with the forward primer 5′-CACTGCCGCATCCTCCTC-3′ and reverse primer 5′-TGCTGTCGCCTTCACCGTTCC-3′. The relative expression levels of the samples were calculated by the 2^−ΔΔCT^ method.

### 2.7. Cell Viability Assay

The cell viability of the NG, 10 mM-glucose, 20 mM-glucose, 30 mM-glucose, and 40 mM-glucose groups was measured using the CCK-8 kit (Beyotime, Shanghai, China). Cells were inoculated in 96-well plates at 100 μL per well containing 5000 cells, and DMEM containing different concentrations of glucose was added to the wells. After incubation, 10 μL of CCK-8 working solution was added to each well and kept for 2 h. Finally, the optical density (OD) value was measured at 450 nm to calculate cell viability.

### 2.8. TdT Mediated dUTP Nick End Labeling (TUNEL)

Apoptosis was detected using the One Step TUNEL Apoptosis Assay Kit (C1086, Beyotime, Shanghai, China). Cell nuclei were stained with DAPI (C1006, Beyotime, Shanghai, China). The experiment was divided into four groups for comparison: NG, HG, HG + OE-NC, and HG + OE-Hhatl. Samples were observed under a confocal laser scanning microscope (LSM 780, Zeiss, Oberkochen, Germany). The number of TUNEL-positive cells was counted and analyzed using ImageJ/Imaris software.

#### 2.8.1. Ca^2+^ Content Determination

Intracellular Ca^2+^ levels in the NG, HG, HG + OE-NC, and HG + OE-Hhatl groups were quantified by Fluo-3 AM fluorescence. rMC-1 cells were incubated with the Ca^2+^ indicator Fluo-3 AM (Beyotime, Shanghai, China) for 1 h at 37°C. Subsequently, rMC-1 cells were washed twice. Images were captured by a Zeiss LSM 780 confocal system (Zeiss, Oberkochen, Germany).

### 2.9. Statistical Analysis

Data analysis was performed with SPSS 21.0 (SPSS, Inc., Chicago, IL, USA) statistical software. The data were normally distributed by the Kolmogorov–Smirnov test, and the results were expressed as mean ± standard deviation. Multiple group comparisons were performed by one-way ANOVA, and two-by-two comparisons after ANOVA were performed by Tukey's multiple comparison test. *p* was a two-sided test. The difference was statistically significant if *p* < 0.05 and statistically highly significant if *p* < 0.01.

## 3. Results

### 3.1. Differential Gene Expression Analysis Revealed Hhatl Was Low-Expressed in Müller Cells of DR Rats

**Figure 1:**
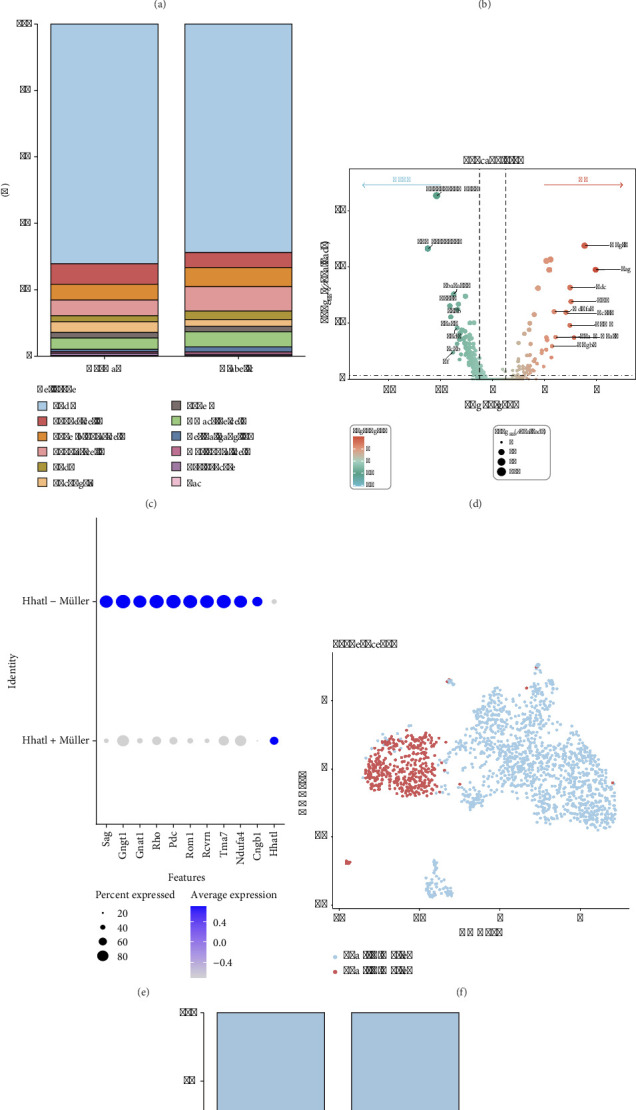
Hhatl was low-expressed in Müller cells of DR rats. (a) Uniform manifold approximation and projection (UMAP) plot of the annotation results of the 12 cell types; (b) bubble plot of key marker genes used to define the 12 cell types; (c) plot of cell type percentage for each group; (d) identification of the DEGs by comparing DR samples with the nondiabetic control sample; (e) plot of the cell subset annotation; (f) UMAP plot of the two subsets: Hhatl^−^ Müller and Hhatl^+^ Müller; (g) plot of the percentage of Hhatl^−^ and Hhatl^+^ Müller cells.

First, this study extracted dataset-related cell marker genes as well as common cell markers for an exhaustive annotation of cell populations, including rod photoreceptors (Rod), Müller cells, endothelial cells (Endo), cone bipolar cells, Amacrine cells, bipolar cells, microglia, retinal ganglion cells, cone photoreceptors (Cone), horizontal cell, erythrocyte, and macrophage (Mac) (Figures 1(a) and 1(b)), and demonstrated the percentage of each cell type in the normal and diabetic group (Figure 1(c)). Given that retinal Müller cells play an important role in maintaining the structural and metabolic functions of the retina [23], the present study focused on the retinal Müller cell population. Figure 1(c) shows a reduced percentage of Müller cells in the diabetic group compared to the Normal group, which caught our attention. Subsequently, selecting the genes via their FC, the top 20 significant DEGs were screened, including 10 low-expressed genes and 10 high-expressed genes (Figure 1(d)). After obtaining Müller cell data, Müller cells were re-annotated by dimensionality reduction clustering analysis, and two cellular subsets were successfully defined as Hhatl^+^ Müller and Hhatl^−^ Müller (Figures 1(e) and 1(f)).  Figure 1(g) displays that in the diabetic group, Hhatl^−^ Müller showed an increasing trend, while the Hhatl^+^ Müller showed the opposite result. This finding suggested that the number ratios of Hhatl^−^ and Hhatl^+^ Müller cells were significantly changed during the course of DR and that this change in the cell number ratio could signal an important effect of Hhatl on DR progression.

### 3.2. Hhatl Inhibited Apoptosis in HG-Treated Müller Cells

**Figure 2:**
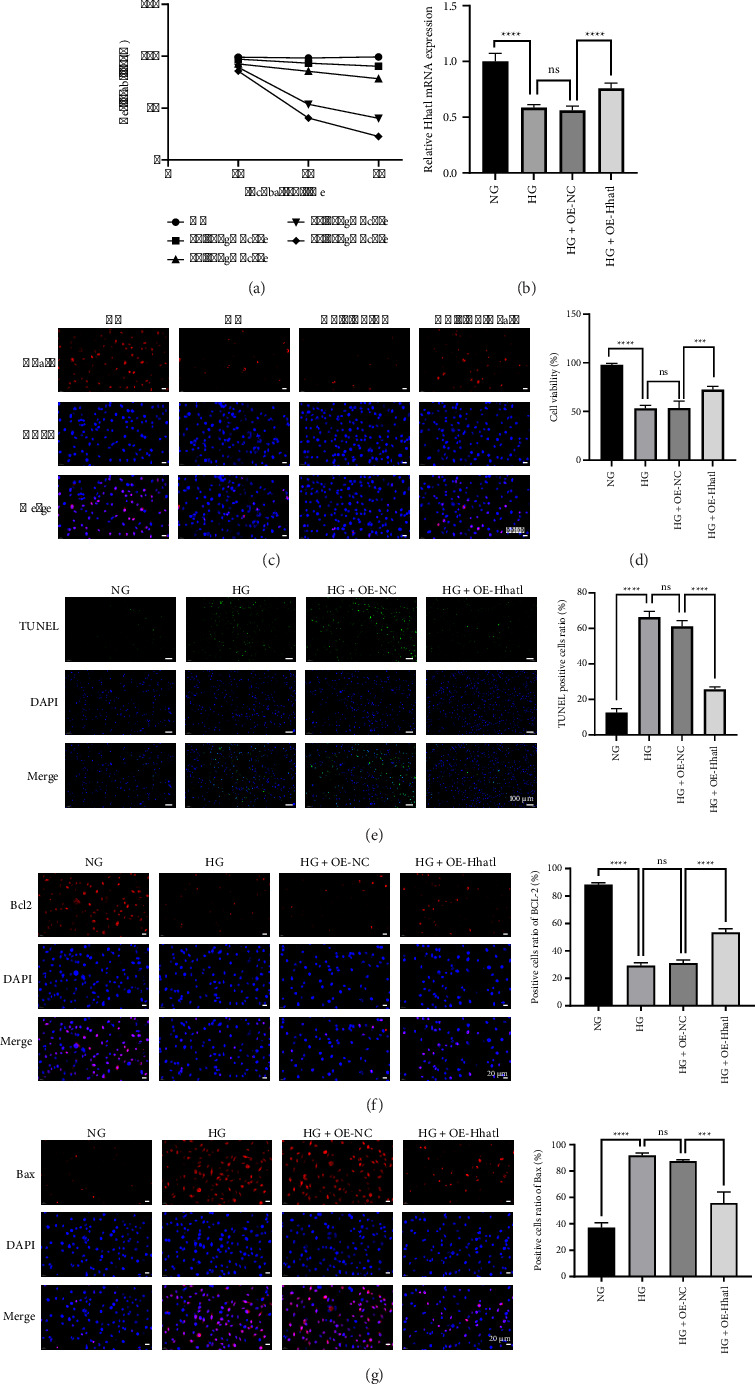
Hhatl inhibited apoptosis in HG-treated Müller cells. (a) Cell viability of rat Müller cells after different glucose concentration and time treatment; (b) RT-qPCR for Hhatl expression in rMC-1 cells. The experiment was independently repeated 6 times (*N* = 6); (c) immunofluorescence for Hhatl expression in rMC-1 cells, with a magnification of 400X (*N* = 3); (d) CCK-8 assay for cell viability (*N* = 6); (e) TUNEL staining for assessment of apoptosis, with a magnification of 100X (*N* = 3); (f, g) immunofluorescence for Bcl2 and Bax levels, with a magnification of 400X (*N* = 3). ^∗^, *p* < 0.05; ^∗∗^, *p* < 0.01; ^∗∗∗^, *p* < 0.001; ^∗∗∗∗^, *p* < 0.0001.

rMC-1 cells were treated with 5.5, 10, 20, 30, and 40 mM glucose to detect cell viability at different time points. As shown in Figure 2(a), cell viability at 48 h in the 30 mM-glucose group was closest to 50%. Thus, this concentration and incubation time were selected for subsequent assays. Next, Hhatl expression in HG-induced rMC-1 cells was examined. RT-qPCR revealed that Hhatl expression in rMC-1 cells was decreased in the HG environment (NG vs. HG, *p* < 0.0001), and transfection with OE-Hhatl upregulated the expression of Hhatl (HG + OE-NC vs. HG + OE-Hhatl, *p* < 0.0001) (Figure 2(b)). The immunofluorescence results were consistent with the RT-qPCR findings (Figure 2(c)). Cell viability and apoptosis were detected by CCK-8 and TUNEL. The results showed that cell viability was decreased (NG vs. HG, *p* < 0.0001) and apoptosis was increased after HG treatment (NG vs. HG, *p* < 0.0001); overexpression of Hhatl restored cell viability (HG + OE-NC vs. HG + OE-Hhatl, *p* < 0.001) and decreased apoptosis to some extent (HG + OE-NC vs. HG + OE-Hhatl, *p* < 0.0001) (Figures 2(d) and 2(e)). Immunofluorescence results confirmed that upregulation of Hhatl did restore B-cell lymphoma 2 (Bcl2) levels and decrease Bcl2-associated X protein (Bax) levels in rMC-1 cells under HG environments (Figures 2(f) and 2(g)). It was verified that HG treatment could decrease Hhatl expression and increase apoptosis in rMC-1 cells; overexpression of Hhatl could inhibit apoptosis and restore cell viability.

### 3.3. Hhatl Reduced Müller Cell Apoptosis by Alleviating ATF6-Related ERS Signaling

**Figure 3:**
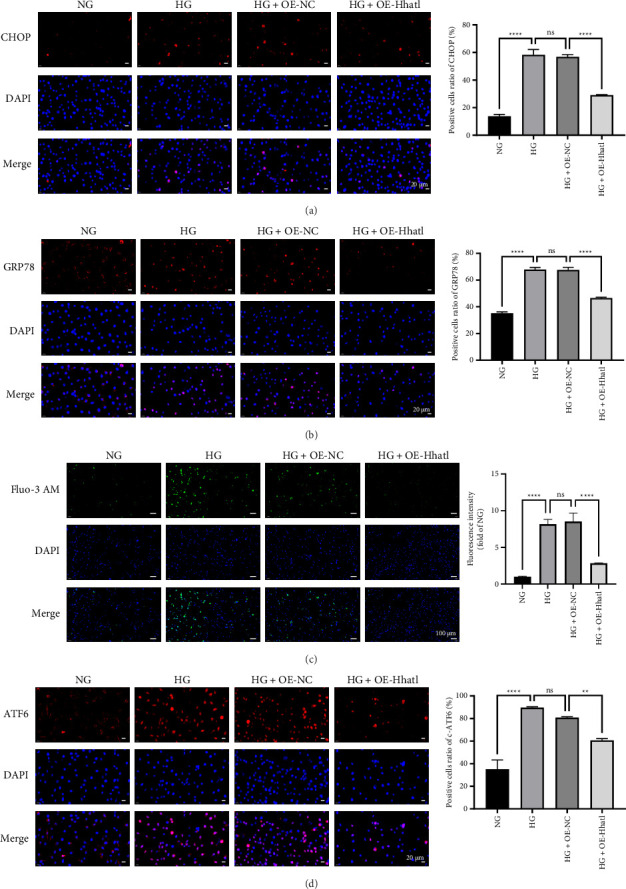
Hhatl reduced Müller cell apoptosis by alleviating ATF6-related ERS signaling. (a, b) Immunofluorescence assay to detect CHOP and GRP78 expression in HG-induced rMC-1 cells, with a magnification of 400X; (c) fluo-3AM fluorescence as cytoplasmic free Ca^2+^ in different groups of rMC-1 cells, and a histogram was used to represent the intracellular Ca^2+^ concentration, with a magnification of 100X; (d) immunofluorescence assay to detect ATF6 expression in HG-treated rMC-1 cells, with a magnification of 400X. ^∗^, *p* < 0.05; ^∗∗^, *p* < 0.01; ^∗∗∗^, *p* < 0.001; ^∗∗∗∗^, *p* < 0.0001.

ERS-related protein expression and intracellular Ca^2+^ concentration were examined to assess whether ERS occurred in HG-induced rMC-1 cells. The assay results showed that C/EBP homologous protein (CHOP) and glucose-regulated protein 78 (GRP78) expression was upregulated, and Ca^2+^ concentration was elevated in HG-treated rMC-1 cells (NG vs. HG, *p* < 0.0001); overexpression of Hhatl reduced CHOP and GRP78 expression in HG-treated rMC-1 cells and lowered Ca^2+^ concentration (HG + OE-NC vs. HG + OE-Hhatl, *p* < 0.0001) (Figures 3(a), 3(b), and 3(c)). The above results confirmed that Hhatl could alleviate ERS in HG-treated Müller cells. Subsequently, this study explored which pathway Hhatl was affecting to alleviate ERS. The expression of ATF6 was increased in HG-treated rMC-1 cells while decreased after overexpression of Hhatl (Figure 3(d)). This result showed that Hhatl could alleviate ATF6-associated ERS signaling.

### 3.4. Hhatl Overexpression Could Alleviate ERS and Apoptosis in ZDF Rats

**Figure 4:**
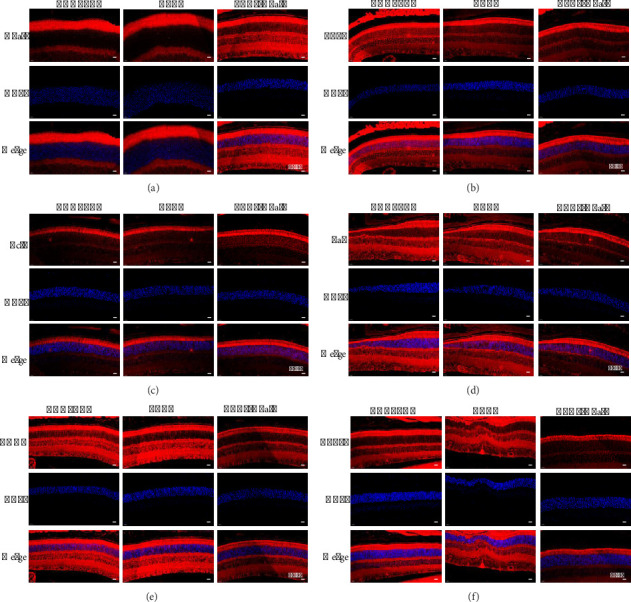
Hhatl overexpression could alleviate ERS and apoptosis in ZDF rats. (a) Hhatl expression in rat eyes, with a magnification of 400X (*n* = 12); (b–f) results of fluorescence immunoreactivity for ATF6, Bcl2, Bax, CHOP, and GRP78, with a magnification of 400X (*n* = 12). ^∗^, *p* < 0.05; ^∗∗^, *p* < 0.01; ^∗∗∗^, *p* < 0.001.

This study further investigated the protective effect of Hhatl on Müller cells in the retina of diabetic rats. First, through immunofluorescence assay detection, Hhatl in the retina of ZDF rats was successfully overexpressed via AAV2-Hhatl (Figure 4(a)). Next, apoptosis and ERS-related proteins were detected. Immunofluorescence results showed that there was no significant difference in ATF6, Bcl2, Bax, CHOP, and GRP78 expression in the control group and the AAV2 group; compared with the AAV2 group, Bcl2 expression was increased, and ATF6, Bax, CHOP, and GRP78 expression was decreased in the AAV2-Hhatl group (Figures 4(b), 4(c), 4(d), 4(e), and 4(f)). From the above results, the in vivo assay results were consistent with the in vitro assay results that retinal ERS and apoptosis could be alleviated by upregulating Hhatl.

## 4. Discussion

The present study, using scRNA-seq, found a decrease in the percentage of retinal Müller cells in DR rats and, for the first time, found the downregulated Hhatl expression in Müller cells in DR by differential gene expression analysis. In addition, two clear cell subsets were successfully defined, namely Hhatl^+^ Müller and Hhatl^−^ Müller. In the DR samples, the number of Hhatl^−^ Müller was increased, while the number of Hhatl^+^ Müller was decreased. Subsequently, whether the decrease in Müller cell number was associated with Hhatl downregulation was verified by cell assays. The results confirmed that Hhatl expression was decreased in HG-treated rMC-1 cells. Overexpression of Hhatl in rMC-1 cells alleviated HG-induced apoptosis and ERS. Mechanistically, overexpression of Hhatl could reduce ATF6 expression, and Hhatl reduces apoptosis in Müller cells by alleviating ATF6-related ERS signaling. Finally, the assay results at the cellular level were further verified by model rats of type II diabetes. ERS and apoptosis-related assay indices were assessed by intravitreal injection of viral vectors overexpressing Hhatl into ZDF rats. Immunofluorescence results showed that ERS and apoptosis appeared in the retina of ZDF rats. In addition, ERS was alleviated, and apoptosis was reduced in the AAV2-Hhatl group. This study revealed a mechanism by which Hhatl protects Müller cells via the ATF6-ERS pathway. However, the cell and animal models used in this study are rats, and their relevance to human biology needs to be further verified through human-derived cells, clinical samples, and translational research. Hhatl is expected to become a novel therapeutic entry point for DR, but its translational application still faces critical challenges of safety and multisystem effects.

ER can sense changes in the cellular microenvironment, coordinate signaling pathways, and regulate cell function and cell survival. When there is an excess of mutant proteins, inflammation, or altered redox status in the body, the function of the ER will be impaired, causing ERS [24]. Mounting studies have looked at treating DR by mitigating ERS. DR can be classified into early and late stages based on clinical symptoms, with the early stage being nonproliferative DR (NPDR) and the late stage being proliferative DR (PDR). Among them, PDR is characterized by the appearance of abnormal neovascularization on the surface of the retina [25]. It has been shown that the ghrelin-GHSR-1a pathway inhibits retinal angiogenesis by attenuating ERS in HG environments [26]. Inflammation is also involved in DR progression. lncRNA MALAT1 in HG-treated human RVECs promotes RVEC angiogenesis and inflammation through the upregulation of ERS, which can be alleviated by knockdown of lncRNA MALAT1 [27]. Xi et al. have found that Sestrin2 could inhibit signal transducer and activator of transcription 3 (STAT3) phosphorylation and ERS and promote autophagy to inhibit ferroptosis in HG-treated ARPE-19 human retinal pigment epithelial cells and DR model mice, thus alleviating DR [28]. ERS is associated with apoptosis in DR. It has been demonstrated that the IP3R1-GRP75-VDAC1 axis promotes the formation of ER-mitochondrial coupling and accelerates Ca^2+^-dependent endothelial cell apoptosis in HG-treated human RVECs (RMECs), and that 4-Phenylbutyric acid (4-PBA), an ERS inhibitor, could alleviate streptozotocin (STZ)-induced retinal dysfunction in DR rats [29]. The present study confirmed that ERS participates in DR progression by regulating apoptosis in Müller cells, further enriching the effect of ERS in DR. However, considering the powerful physiological functions of Müller cells, whether Hhatl has any effect on other aspects of Müller cells, such as secretory functions, is not yet known, and thus an in-depth exploration of Hhatl is warranted in subsequent studies in the future.

Hedgehog (Hh) proteins are members of the family of a small number of secreted signaling proteins. The Hh signaling pathway plays a crucial role in regulating animal development and maintaining tissue homeostasis [30]. Abnormalities of the Hh pathway have been confirmed to be associated with various human diseases, including rheumatic diseases, cancer, and inflammatory bowel disease [31–33]. Hhatl and Hhat are core molecules that regulate the Hh signaling pathway. Hhat can catalyze the palmitoylation of Hh signaling, while Hhatl can inhibit this palmitoylation. Together, they determine the strength and biological effects of Hh signaling through palmitoylation modification and negative feedback mechanisms [34]. Currently, there is little research on Hhatl. Early researchers have revealed that Hhatl and Hhat are homologous to *Saccharomyces cerevisiae* glycerol uptake/transporter 1 (Gup1) and Gup2, respectively. Furthermore, Gup1 is associated with multiple biological functions [35]. Studies have shown that in *Saccharomyces cerevisiae* mitochondria, voltage-dependent anion channel 1 (Por1/yVDAC1) physically interacts with Gup1, regulating cell wall integrity and the process of programmed cell death [36]. A recent study has demonstrated that Hhatl expression is downregulated in patients with hypertrophic cardiomyopathy. The loss of Hhatl will activate the Sonic hedgehog (SHH) signaling pathway and promote the expression of hypertrophy markers, ROS production, and mitochondrial dysfunction, ultimately leading to myocardial hypertrophy [20]. Additionally, a contemporaneous study has also indicated that knockdown of Hhatl exacerbates ER stress and cell apoptosis [21]. These studies on the function of Hhatl are consistent with our experimental results. In pathological environments, Hhatl expression is downregulated, and this reduction may be related to cell apoptosis and ER stress.

ERS has three classical activation pathways, namely the protein kinase R-like ER kinase (PERK)/CHOP/ATF4 pathway, the ATF6 pathway, and the IRE1/XBP1 pathway [37]. lncRNA GAS5 expression was reduced in HG-treated retinal epithelial cells, and upregulation of lncRNA GAS5 can reduce the phosphorylation levels of PERK and eukaryotic initiation factor 2*α* (eIF2*α*) in ARPE-19 cells under HG environments, alleviating the ERS [38]. Wu et al. have identified transcription factor 7-like 2 (TCF7L2) as a susceptibility gene for DR. TCF7L2 expression was significantly elevated in the retina of diabetic model mice and promoted ERS in DR, and knockdown of TCF7L2 in human induced pluripotent stem cells (hiPSCs)-derived retinal progenitor cells (RPCs) significantly inhibited ATF6-related ERS signaling [39]. The results of the present study showed that Hhatl regulated ERS in Müller cells by affecting the ATF6 pathway. Ca^2+^ is involved in a wide range of cellular physiological activities, and the ER is a major source of Ca^2+^ for intracellular signaling [40]. It has been demonstrated that HG can increase intracellular Ca^2+^ concentration, and downregulation of transient receptor potential cation channel 6 (TRPC6) expression can decrease intracellular Ca^2+^ concentration and reduce ROS production in rat Müller cells, and that silencing of TRPC6 under HG conditions can prevent Müller cell apoptosis and decrease secretion of interleukin-6 (IL-6) and VEGF. This finding suggests that apoptosis and neurovascular changes in retinal Müller cells in DR can be counteracted by decreasing intracellular Ca^2+^ concentration [41]. Overexpression of Hhatl was also found to reduce intracellular Ca^2+^ concentration in our assays. However, the present study only briefly explored the regulation of ERS by Hhatl through the ATF6 signaling pathway. Whether Hhatl interacts with other molecules, as well as the existence of post-transcriptional or epigenetic regulatory mechanisms, remains unknown. These questions will be the direction of our in-depth research in the future.

In summary, scRNA-seq analysis combined with cellular and animal assays demonstrated that upregulated Hhatl plays a protective role by alleviating ATF6-associated ERS signaling in HG-treated rMC-1 cells and ZDF model rats, which uncovers a potential therapeutic target for DR treatment. This study only verified the effects of Hhatl on ER and apoptosis in ZDF rats. ZDF rats mainly simulate type 2 diabetes-related DR, but it remains unclear whether they cover the PDR stage (such as neovascularization). The pathological differences between the model and human DR may lead to biased results. Therefore, further verification in more species is required. In the animal experiment section, we used AAV2 to increase Hhatl expression in the retinas of ZDF rats, but this approach lacked cell type specificity. In subsequent studies, we will employ AAV vectors carrying Müller cell-specific promoters to further regulate the expression of Hhatl in Müller cells specifically. This will allow for more precise verification of its mechanism in DR and reduce the potential impact of nonspecific infection on the results. In addition, it is not clear whether Hhatl plays different roles in DR through multiple pathways. Therefore, further investigation should be conducted in the future for a more comprehensive understanding of the functions of Hhatl.

## Data Availability

The data that support the findings of this study are available from the corresponding author upon reasonable request.
